# Medical Notes and Comments

**Published:** 1878-06

**Authors:** 


### Medical Notes and Comments.

The Medical Graduates of 1878 number 1,723, Jefferson Medical College heading the list with 203, while the Women's Hospital Medical College at Chicago, brings up the rear with only 7. Over thirty regular medical colleges now exist within the United States, and nine of these, each graduated over one hundred young men fully equipped to practice the noble art of healing. Thus the ranks of the profession are to be kept at the full normal standard.——A Circular dated April 27th, from Surgeon General JOHN M. WOODWORTH, directs the Medical Officers of the U. S. Marine Hospital Service to make use of the metric system of weight and measures for all Medical and Pharmacal purposes. Rules are given for the ready conversion of weights and measures now in use into the metric system. It would be an advance in the right direction if this system could be brought into general favor.——AMPUTATION OF THE CERVIX UTERI, by W. H. WATHEN, M. D. Reprint from the May No. *Richmond and Louisville Medical Journal.* This paper gives a history of the operation and the conditions in which the operation is indicated, with the results and dangers attending it. A scissors invented by the author, is described and figured. It is called "the cervix scissors," and is claimed to combine the advantages of all the other instruments in use. He also figures a new double tenaculum forcep to hold the cervix while using the scissors. He concludes with a report of the first operation with his cervix scissors.——The PHYSIOLOGY AND PSYCHOLOGY OF RELIGIOUS REVIVALS, by Z. Collins McElroy, M. D. Reprint from Cincinnati *Lancet and Observer.* The author defines 'psychology, as referring to mind phenomena, the function of the structural arrangement of a part of the brain matter of a human being,' and upon this thought he endeavors to build a system which, if carried to completion, would account for all our intellectual processes. He has given in brief what has been wrought out in full many times before, but as the subject matter has little or no bearing upon medicine, we will say no more about the pamphlet.——Dr. E. H. COOVER suggests *silicate of Soda* as a substitute for plaster of Paris in the Treatment of Spinal Diseases and Curvatures, Reprint from *Medical and Surgical Reporter* of April 13, 1878. It is lighter, acts nearly as quickly, does not crumble, and may be readily reapplied.——BALMANNO SQUIRE has invented a new *Linear Multiple Scarifier* for the treatment of various lesions of the skin.——COST OF KEEPING THE PAUPERS OF THE EIGHTH JUDICIAL DISTRICT.—Average weekly cost for each pauper in the District as a whole, $1.45,—in Allegany Co., $1.27,—Cattaraugus Co. $1.14, Chautauqua Co. $0.90,—Erie Co. $1.70,—Genesee Co. $1.05,—Niagara Co. $1.58,—Orleans Co. $1.67,—Wyoming Co. $1.15. Chautauqua county supported two hundred and ten paupers in the County Poor House for a little over $10,000, while Erie county required $60,000 for the support of seven hundred. The average of the District by counties would be about $1.31 as given in our last, but this is not the true average cost which is as above $1.45. —— Objections to the Use of Carbolic Acid in the Treatment of Piles, by J. M. MATTHEWS, M. D., Reprint from the *American Medical Bi-Weekly* April 28, 1878. The writer gives as his conclusions: "1st. Use the acid only in the smallest tumors. 2d. Should it be used in a large tumor inject once only in one portion, and wait several days, and then inject another portion. 3d. Use the smallest amount possible in injecting, say one to three drops of the mixture of sweet oil and carbolic acid equal parts, or a stronger solution."——We most heartily agree with the following advice to authors, which we find in the *Richmond and Louisville Medical Journal.* "Pamphlets in large numbers are constantly received by the Editors of the Medical Press, and these gentlemen would be glad to notice them, but as a rule this is difficult and involving much labor, for the simple reason that the authors have failed to adopt a device not only simple and easy of execution, but one which would be welcome to every reader, viz., *the closing of the article by giving a summary of the views or arguments advanced.*" Most pamphlets would receive more notice if this sensible advice should be adopted.——A NEW OBSTETRICAL JOURNAL will issue from Cincinnati in July next by E. B. STEVENS, many years editor of the Cincinnati *Lancet and Observer.* We wish him success in his new enterprise.——We abstract the following from the proceedings of the State Society, and sincerely hope that the Hot Springs physicians will take warning and be more discreet and ethical in the future. Their actions, which have been promptly and justly rebuked, were an insult to the profession.

"*Resolved*, That no member of the Hot Springs and Garland County Medical Society be allowed to register, or delegate therefrom be admitted at this meeting of the Society."

*Resolved*, That Drs. P. H. Ellsworth, O. A. Hobson, G. W. Lawrence, S. W. Franklin and E. A. Shippey, *be expelled* from all the rights, privileges and immunities of the State Medical Society of Arkansas." C. A. W.

